# Computer Tomography-Guided Pulmonary Microcoil Insertion for the Localisation of Small Pulmonary Nodules at the Time of Pulmonary Resection

**DOI:** 10.7759/cureus.98334

**Published:** 2025-12-02

**Authors:** Edward Wang, Samantha Ellis, Loret Kotevski, Christopher Merry, Miranda Siemienowicz, Julian Gooi

**Affiliations:** 1 Cardiothoracic Surgery and Transplantation, The Alfred Hospital, Melbourne, AUS; 2 Radiology, The Alfred Hospital, Melbourne, AUS; 3 Surgical Services - Operating Suite Nursing, The Alfred Hospital, Melbourne, AUS; 4 Northern Imaging Victoria, Northern Health, Melbourne, AUS

**Keywords:** ct guided microcoil insertion, lung cancer screening, microcoil, minimally invasive lung resection, pulmonary nodule, small pulmonary nodule

## Abstract

Background

Small and ground glass pulmonary nodules pose a surgical and diagnostic challenge, with an increase in identification expected with the roll-out of lung cancer screening in Australia. Accurate preoperative localisation of small pulmonary nodules can be a useful adjunct to lung resection in nodules that are not palpable. Various methods have been trialled including methylene blue dye injection and hook-wire placement; however, these are limited by dye diffusion, wire dislodgement, and patient discomfort. CT-guided insertion of a microcoil is a technique of localisation that provides a stable and visible marker on X-ray, improving intraoperative identification. This study reports an Australian institutional experience with this technique and evaluates its safety and efficacy.

Methods

A retrospective review of all patients undergoing CT-guided pulmonary microcoil localisation followed by video-assisted thoracoscopic surgery (VATS) was conducted at the Alfred Hospital, Melbourne. A case series of 19 patients from 2021 - 2025 was identified and data on technical success, complications, operative outcomes, and pathology were collected.

Results

The mean patient age was 63.5 years, with a slight male predominance (n=11, 57.8%). Indications for lung resection included pulmonary metastasectomy (n=12, 63.2%) and primary lung adenocarcinoma (n=7, 36.8%). The indication for microcoil guidance was a deep nodule in 36.8% (n=7) of cases and in 63.1% (n=12) of cases an impalpable nodule due to small size or ground glass appearance. The technical success of microcoil placement was 94.3% (n=18), with one displaced microcoil early in the series. No pneumothorax requiring intervention occurred and no major radiological complications from microcoil placement seen in the series. All lesions were successfully resected, with clear histologic margins and no major surgical complications. The mean chest drain duration was 3.4 days and mean hospital stay 4.6 days.

Conclusions

CT-guided pulmonary microcoil insertion is a safe, reliable, and reproducible technique for intraoperative identification of small or impalpable pulmonary nodules. It addresses some of the limitations of previous localisation methods and demonstrates excellent outcomes in an Australian setting. Future developments may include indocyanine green-coated microcoils for fluorescence guidance during robotic-assisted thoracic surgery.

## Introduction

Small pulmonary nodules pose a diagnostic and surgical challenge. The adoption of low-dose computed tomography (LDCT) for lung cancer screening has led to a marked increase in the detection of small pulmonary nodules [1,2]. Many of these lesions - particularly those that are subcentimetre, ground-glass, or located deep within the parenchyma - may not be palpable or visible. At the time of video-assisted thoracoscopic (VAT) lung resection, intraoperative identification of these nodules may be difficult or impossible without prior localisation [3,4]. Failure to accurately identify the target lesion can result in incomplete resection, unnecessary conversion to lobectomy, prolonged operative times or conversion to open thoracotomy, thereby compromising patient outcomes [5]. Consequently, reliable preoperative localisation has become an essential adjunctive technique for thoracic surgeons during minimally invasive pulmonary resection.

A variety of localisation techniques have been developed and some trialled at the Alfred Hospital. These include hook wire insertion, methylene blue dye injection, lipiodol, and radio-isotopic markers, each with distinct advantages and limitations in terms of accuracy, safety, and practicality [6,7]. Among these, CT-guided microcoil localisation has gained prominence as one of the most reliable and reproducible methods, with other institutions publishing technical success rates exceeding 95% and low complication rates [8-11]. The microcoil serves as a stable intrapulmonary marker, providing clear fluoroscopic and visual guidance while minimising the risks of dislodgement and patient discomfort compared with rigid hook-wire systems [12,13]. Larger contemporary series have confirmed its safety and versatility in both single and multiple nodule settings [14,15].

This study presents our institutional experience with CT-guided pulmonary microcoil localisation for VAT surgery (VATS) resection at the Alfred Hospital in Melbourne, Australia. We evaluate the safety, technical success, and postoperative outcomes associated with this technique and discuss its role in the evolving paradigm of minimally invasive thoracic surgery. By contributing local data to the global literature, this study aims to reinforce the feasibility and reproducibility of microcoil localisation within an Australian thoracic surgical context.

## Materials and methods

A retrospective review was conducted of all patients who underwent CT-guided pulmonary microcoil insertion for intraoperative localisation prior to VATS pulmonary resection at the Alfred Hospital, Melbourne, between 2021 and 2025. Early localisation experience at our institution employed hook wires and methylene blue injection; however, these methods were abandoned due to issues including wire displacement, pleural pain, and dye diffusion. Following lung cancer multidisciplinary discussion, the microcoil technique was adopted for nodules that were small, deep, or of ground-glass morphology (following multidisciplinary lung cancer meeting discussion).

**Figure 1:**
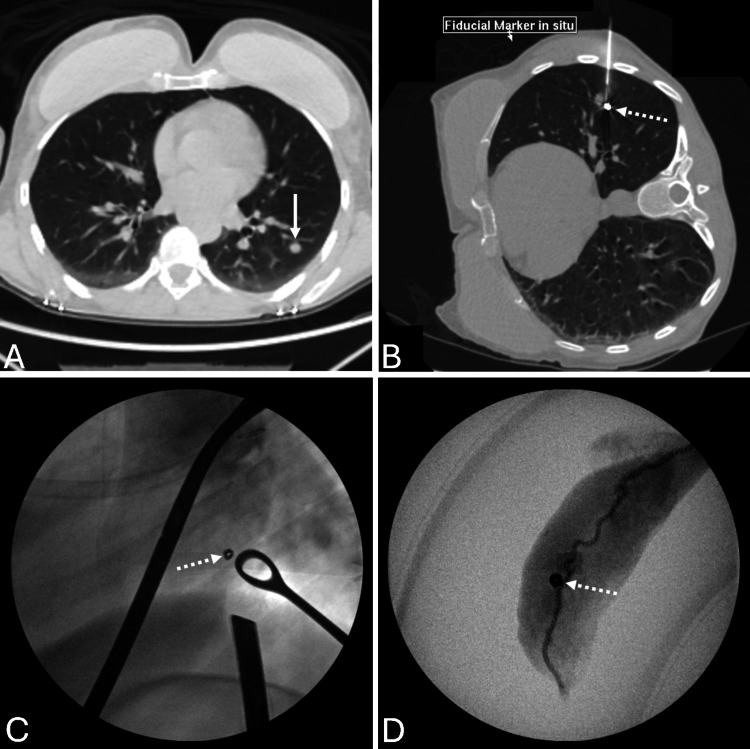
Radiology images of the pulmonary microcoil insertion and pulmonary resection A: Pre-coil insertion CT demonstrating pulmonary nodule B: CT guided insertion of Vortx microcoil in the nodule C: Intraoperative fluoroscopy with the microcoil identified, with lung grasper and stapler being positioned D: Back-table fluoroscopy confirming microcoil within the resected specimen

**Figure 2:**
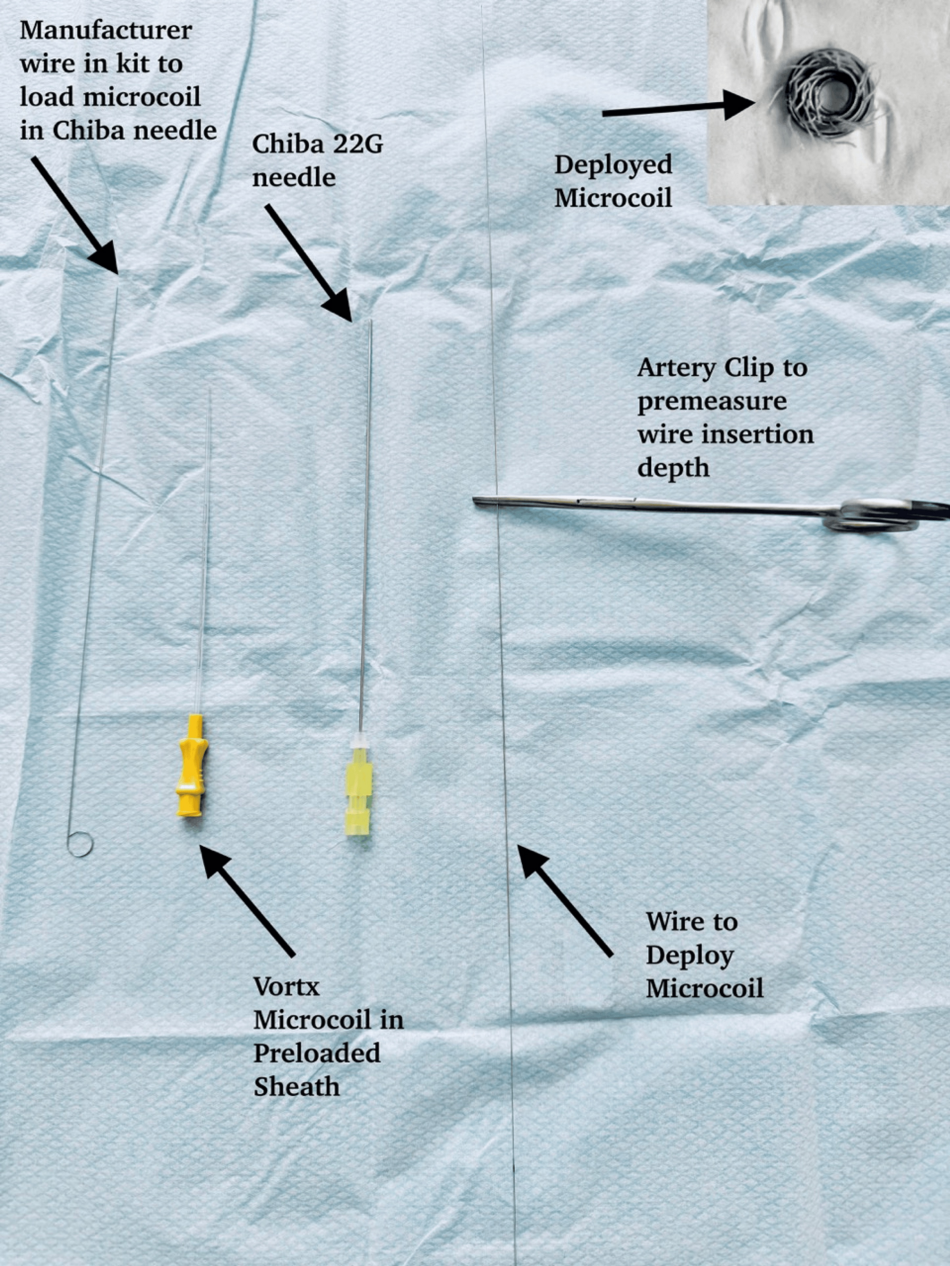
CT-guided pulmonary microcoil insertion equipment set-up

The CT-guided pulmonary microcoil insertion is performed under local anaesthetic by one of two subspecialist thoracic interventional radiologists. In each case, pre-procedural CT imaging is reviewed to select a safe trajectory avoiding vessels, fissures, and airways as illustrated in Figure 1. A 5 × 5.5 mm VortX Diamond 18 pushable microcoil (Boston Scientific, Marlborough, MA, USA) is preloaded into a 20G 15 cm Chiba needle. The equipment used is displayed in Figure 2. Under intermittent low-dose CT “smart-step” guidance, the needle is advanced stepwise to the target area. Once appropriately positioned, the microcoil is deployed by inserting the micropuncture wire into the needle hub and gently advancing the preloaded microcoil into the pulmonary parenchyma. A non-tailed intrapulmonary technique is employed, with the microcoil positioned at or adjacent to the deep margin of the lesion according to surgeon preference. A completion CT scan confirms microcoil position and excludes immediate complications.

VAT resection is then performed on the same day by one of two subspecialist thoracic surgeons. Lobar anatomy and visual evidence of pleural surface puncture is used to identify likely position of the microcoil, and then intraoperative X-ray is used to identify the microcoil and guide stapler positioning to ensure adequate margins. Following resection, the excised specimen is imaged with X-ray to confirm the presence of the microcoil as seen in Figure 1.

Data were obtained through retrospective chart review, including demographics, smoking history, pulmonary function, procedural parameters, complications, and histopathological outcomes. Follow-up included assessment of postoperative complications and mortality. Ethics approval was obtained from the Alfred Hospital Human Research Ethics Committee (Project ID: 475/25).

## Results

Between 2021 and 2025, a total of 19 patients underwent CT-guided pulmonary microcoil insertion for intraoperative localisation of small pulmonary nodules. The patient characteristics are outlined in Table 1. The mean patient age was 63.5 years, with a slight male predominance (n=11, 57.8% male). The mean BMI was 24.8 kg/m², and 47.4% (n=9) were current or former smokers. The median Eastern Cooperative Oncology Group (ECOG) performance status was 0.

**Table 1:** Patient characteristics

Characteristic	Value
		Number of Patients	19
Age (years)			
Median (Q1 – Q3)	67 (54 - 72)
Range	37 – 85
Gender	
Female (n, %)	8 (42.1%)
Male (n, %)	11 (57.9%)
Smoking Status	
Smoker (n, %)	9 (47.4%)
Non-smoker (n, %)	10 (52.6%)
BMI	
Mean (STD)	24.8 (4.0)

The pulmonary nodule characteristics are detailed in Table 2. The most common indication for resection was pulmonary metastasectomy (n=12, 63.2%), followed by primary lung adenocarcinoma (n=7, 36.8%). The mean consolidation-to-tumour ratio amongst the adenocarcinoma cases was 0.11, with more than half being pure ground-glass lesions. The indication for microcoil localisation was reviewed and in 36.8% (n=7) was due to the lesion being deep in parenchyma with a mean depth of 1.9cm. In the remaining 12 patients (63.1%), the indication for microcoil localisation was an impalpable nodule, either due to being subcentimeter or ground glass predominant lesions.

**Table 2:** Nodule characteristics

Characteristic	Value
		Number of Patients	19
Nodule Type			
Ground glass (n, %)	4 (21.1%)
Part solid (n, %)	4 (21.1%)
Solid (n, %)	11 (57.9%)
Nodule Size (mm)	
Median (Q1 – Q3)	7 (6 – 13)
Nodule Depth (mm)	
Mean (STD)	10.4 (8.7)
Nodule Location	
Upper Lobe (n, %)	6 (31.6%)
Middle Lobe (n, %)	1 (5.2%)
Lower Lobe (n, %)	12 (61.2%)
Nodule Growth	
Growth (n, %)	14 (73.7%)
Nil growth (n, %)	5 (26.3%)
Nodule Pathology	
Lung Adenocarcinoma (n, %)	6 (31.6%)
Lung Adenocarcinoma in situ (n, %)	2 (10.5%)
Metastatic Melanoma (n, %)	4 (21.1%)
Metastatic Colorectal Carcinoma (n, %)	4 (21.1%)
Metastatic Renal Cell Carcinoma (n, %)	1 (5.3%)
Benign Pulmonary Granuloma (n, %)	1 (5.3%)

The technical success rate for microcoil placement was 94.3% (n=18). Only one microcoil displacement occurred early in the series (5.2%) with the microcoil displaced into the pleural space. The mean procedural duration was 52 minutes, demonstrating improved efficiency as experience accrued, with procedure times reducing to 20 minutes in later cases. No major microcoil insertion-related complications occurred. Small, asymptomatic pneumothoraces were seen in 11 (57.9%) patients; none required drain insertion. One unexpected complication involved an issue with coil deployment. During the initial attempt to place the coil, the Vortx microcoil became fixed in the Chiba needle. Unfortunately, a small pneumothorax developed, precluding immediate re-attempt. The procedure was abandoned and completed successfully at a later date. 

Eighty-nine percent (n=17) of patients underwent same-day VATS resection, and all except one achieved successful localisation and resection of the target lesion. Procedures performed included wedge resection (n=13, 72.2%), segmentectomy (n=4, 22.2%), and lobectomy (n=1, 5.5%). The case of microcoil localisation facilitating lobectomy was a patient with absent fissures where the microcoil allowed stapler placement with adequate margins. No cases required conversion to open thoracotomy and no cases required conversion to a larger than planned resection due to failure to localise the pulmonary nodule. The mean operative time was 76 minutes. At histopathology, clear surgical margins were achieved in all resections, with a mean lesion size of 10.5 mm and mean margin of 10.6 mm. The benign resected rate was 5% (n=1). Pathology included adenocarcinoma (n=6, 31.6%), adenocarcinoma in situ (n=2, 10.5%), metastatic melanoma (n=4, 21.1%), metastatic colorectal carcinoma (n=4, 21.1%), metastatic renal cell carcinoma (n=1, 5.3%), and benign granuloma (n=1, 5.3%).

Postoperative outcomes were favourable, with no surgical mortality or major morbidity. Minor surgical complications included uncontrolled postoperative pain (n=2, 10.5%), subcutaneous emphysema (n=1, 5.2%), and prolonged air leak (>7 days, n=2, 10.5%). The mean chest drain duration was 3.4 days, and the mean hospital stay was 4.6 days.

## Discussion

This study adds to the current evidence that CT-guided pulmonary microcoil localisation is a safe, effective, and reproducible technique for intraoperative identification of small or impalpable pulmonary nodules during VATS lung resection. The technical success rate of 94.3% and absence of major complications are consistent with large international series reporting success rates exceeding 95% and low morbidity [1-3,8-12]. Microcoils provide stable and identifiable intraoperative landmarks, permitting localisation in surgery and helping achieve no conversion to open thoracotomy, preservation of lung parenchyma and successful resection with clear margins in all the cases in this series. With the expected surge in identification of small and ground glass nodules through the rollout of lung cancer screening in Australia, this localisation technique is a practical and effective tool for the modern multidisciplinary thoracic service in Australia.

Before adopting the microcoil method, our institution trialled methylene blue dye and hook-wire techniques. Both approaches presented technical issues. Methylene blue was prone to diffusion resulting in some unreliability of intraoperative localisation. Hook wires troubled patients with pain due to the retained wire through the chest wall and the risk of dislodgement, as previously reported [6,7]. These drawbacks led to our transition to microcoil localisation, which offers improved stability, reduced discomfort, and reliable intraoperative fluoroscopic confirmation. Our findings mirror those from major studies confirming that microcoil localisation is among the most consistent, safe and workable preoperative marking techniques available [8-15].

This study is limited by its retrospective design and small sample size. It represents a single-centre experience, and all procedures were performed by a small group of subspecialist radiologists and surgeons, introducing potential operator bias. Nonetheless, our results demonstrate that microcoil localisation is achievable, safe, and reproducible within an Australian tertiary setting, yielding outcomes comparable to leading international institutions [8,11,13].

Future research should explore enhancements to this technique. Our thoracic surgery unit’s move to adopt robotic-assisted resection provides opportunity for evolution. In particular, coating microcoils with indocyanine green (ICG) could allow Firefly near-infrared fluorescence localisation during robotic-assisted thoracic surgery (RATS). This multimodal approach could improve efficiency by allowing localisation without the need for moving in a bulky X-ray arm in for intraoperative guidance [9]. Multicentre, prospective studies will be essential to validate such innovations, assess their cost-effectiveness, and evaluate their impact on oncologic outcomes.

## Conclusions

CT-guided pulmonary microcoil localisation is a safe, reproducible, and effective adjunct for VATS resection of small or impalpable pulmonary nodules. In this Australian institutional experience, high localisation success and minimal morbidity were achieved. With the adoption of lung cancer screening in Australia and expected surge in identification of small and ground glass nodules, this localisation technique presents an excellent tool for the management of these patients.
